# Supplementary material for: Dual-Responsive Supramolecular Chiral Assemblies from Amphiphilic Dendronized Tetraphenylethylenes
Source: Molecules. 2023 Sep 12;28(18):6580. doi: 10.3390/molecules28186580 (PMC10537776; doi:10.3390/molecules28186580)
Supplement: Supplementary file 1 [file molecules-28-06580-s001.zip › molecules-2581143-supplementary.pdf]

Supplementary Materials

Dual-Responsive Supramolecular Chiral Assemblies from Amphiphilic Dendronized Tetraphenylethylenes

Jianan Zhang, Xueting Lu, Wen Li* and Afang Zhang*

International Joint Laboratory of Biomimetic and Smart Polymers, School of Materials Science and Engineering, Shanghai University, Materials Building Room 447, Nanchen Street 333, Shanghai 200444, China. E-mails: wli@shu.edu.cn (W.L.), azhang@shu.edu.cn (A.Z.)

Table of Contents

Figure S1. ^{1}H NMR spectrum of **Boc-PO(Ms)G-OMe** in d_6-DMSO S4

Figure S2. ^{13}C NMR spectrum of **Boc-PO(Ms)G-OMe** in d_6-DMSO S4

Figure S3. ^{1}H NMR spectrum of **Boc-PO(N$_3$)G-OMe** in d_6-DMSO S5

Figure S4. ^{13}C NMR spectrum of **Boc-PO(N$_3$)G-OMe** in d_6-DMSO S5

Figure S5. ^{1}H NMR spectrum of **Boc-PO(N$_3$)G-OH** in d_6-DMSO.. S6

Figure S6. ^{13}C NMR spectrum of **Boc-PO(N$_3$)G-OH** in d_6-DMSO.. S6

Figure S7. ^{1}H NMR spectrum of **Boc-PO(N$_3$)G-TPE** in d_6-DMSO S7

Figure S8. ^{13}C NMR spectrum of **Boc-PO(N$_3$)G-TPE** in d_6-DMSO S7

Figure S9. ^{1}H NMR spectrum of **Boc-PO(Me)G-TPE** in d_6-DMSO...................................... S8

Figure S10. ^{13}C NMR spectrum of **Boc-PO(Me)G-TPE** in d_6-DMSO.................................... S8

Figure S11. ^{1}H NMR spectrum of **Boc-PO(Et)G-TPE** in d_6-DMSO S9

Figure S12. ^{13}C NMR spectrum of **Boc-PO(Et)G-TPE** in d_6-DMSO S9

Figure S13. ^{1}H NMR spectrum of **H-PO(Me)G-TPE** in d_6-DMSO S10

Figure S14. ^{13}C NMR spectrum of **H-PO(Me)G-TPE** in d_6-DMSO S10

Figure S15. ^{1}H NMR spectrum of **H-PO(Et)G-TPE** in d_6-DMSO...S11

Figure S16. ^{13}C NMR spectrum of **H-PO(Et)G-TPE** in d_6-DMSO...S11

Figure S17. ESI-MS (pos. mode, DCM) of **Boc-PO(Ms)G-OMe**. S12

Figure S18. ESI-MS (pos. mode, DCM) of **Boc-PO(N$_3$)G-OMe**. S12

Figure S19. ESI-MS (pos. mode, DCM) of **Boc-PO(N$_3$)G-OH**. S13

Figure S20. ESI-MS (pos. mode, DCM) of **Boc-PO(N$_3$)G-TPE**. S13

Figure S21. ESI-MS (pos. mode, DCM) of **Boc-PO(Me)G-TPE**...S14

Figure S22. ESI-MS (pos. mode, DCM) of **Boc-PO(Et)G-TPE**. S14

Figure S23. ESI-MS (pos. mode, DCM) of **H-PO(Me)G-TPE**. S15

Figure S24. ESI-MS (pos. mode, DCM) of **H-PO(Et)G-TPE** S15

Figure S25. NOESY spectra of **H-PO(Me)G-TPE** in D_2O at pH 4 (a), pH 5 (b), pH 6 (c), and pH 7 (d). T = 25 °C. C = 1.0 mg·mL^{-1}.. S16

Figure S26. NOESY spectra of **H-PO(Et)G-TPE** in D_2O at pH 1 (a), pH 3 (b), pH 5 (c), and pH 6 (d). T = 25 °C. C = 1.0 mg·mL^{-1}. S16
Figure S27. Temperature-varied FL spectra of **H-PO(Me)G-TPE** in aqueous solutions at pH 4 (a), pH 5 (b), pH 6 (c), and pH 7 (d). C = 0.2 mg·mL^{-1}, λ_{ex} = 325 nm. S17
Figure S28. Temperature-varied FL spectra of **H-PO(Et)G-TPE** in aqueous solution at pH 1 (a), pH 2 (b), pH 3 (c), pH 4 (d), pH 5 (e), and pH 6 (f). C = 0.2 mg·mL^{-1}, λ_{ex} = 325 nm. S17
Figure S29. ^{1}H NMR spectra of **H-PO(Me)G-TPE** (a) and **H-PO(Et)G-TPE** (b) in D_2O at different temperature. pH = 6, C = 1.0 mg·mL^{-1}. S18
Figure S30. NOESY spectra of **H-PO(Me)G-TPE** in D_2O at 25 °C (a), 60 °C (b), and 75 °C (c). pH = 6. C = 1.0 mg·mL^{-1}. S18
Figure S31. NOESY spectra of **H-PO(Et)G-TPE** in D_2O at 10 °C (a), 25 °C (b), 45 °C (c), and 70 °C (d). pH = 6 .... S19
Figure S32. Temperature-varied CD and UV spectra of the assembly from **H-PO(Me)G-TPE** in aqueous solutions at pH 4 (a), pH 5 (b), pH 6 (c), and pH 7 (d) S19
Figure S33. Temperature-varied CD and UV spectra of the assembly from **H-PO(Et)G-TPE** in aqueous solutions at pH 1 (a), pH 2 (b), pH 3 (c), pH 4 (d), pH 5 (e), and pH 6 (f) S20

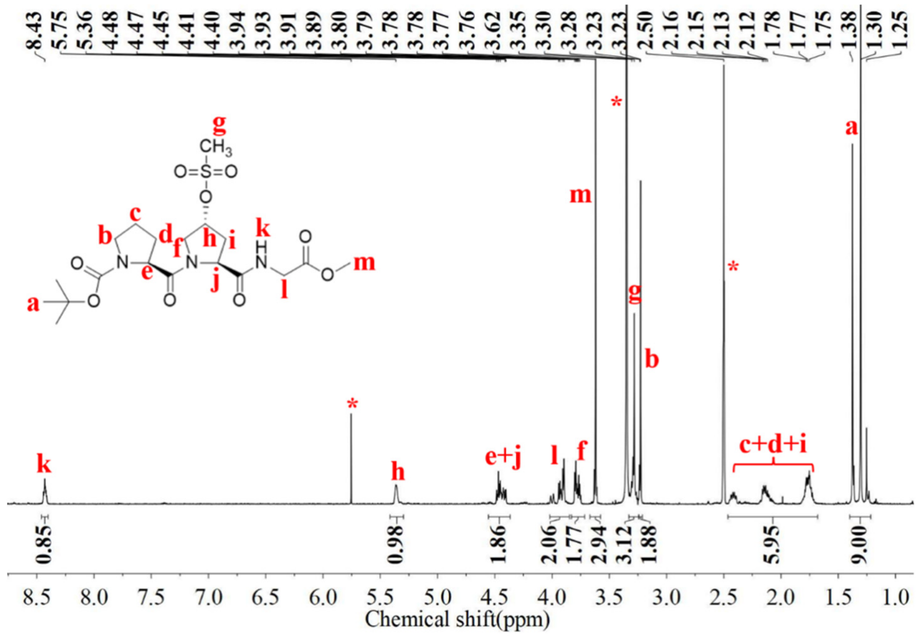

Figure S1. ^{1}H NMR spectrum of **Boc-PO(Ms)G-OMe** in *d*6-DMSO. The solvent peak is marked with asterisk (*).

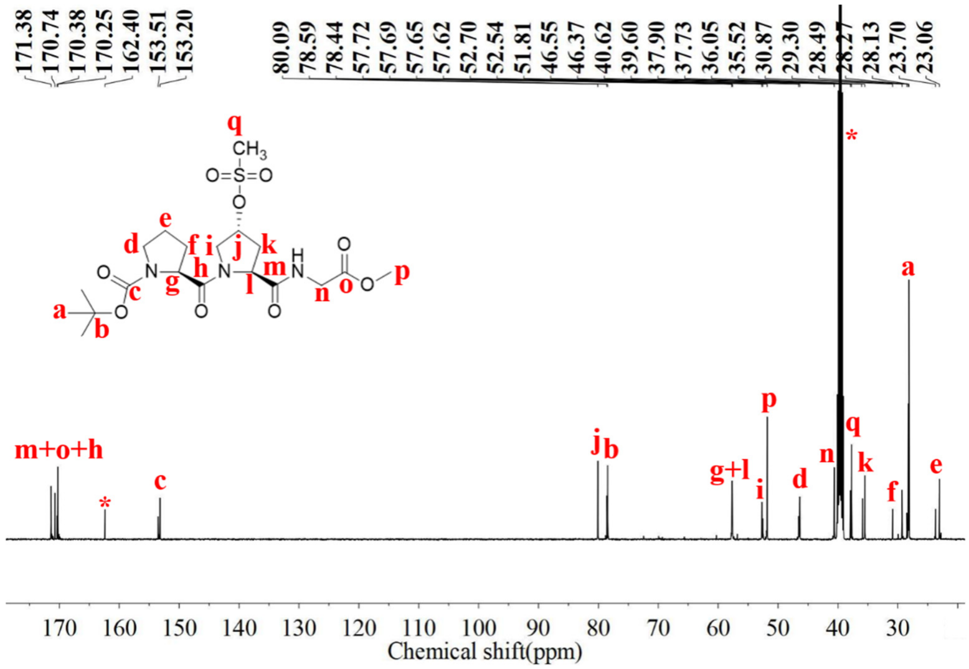

Figure S2. ^{13}C NMR spectrum of **Boc-PO(Ms)G-OMe** in *d*6-DMSO. The solvent peak is marked with asterisk (*).

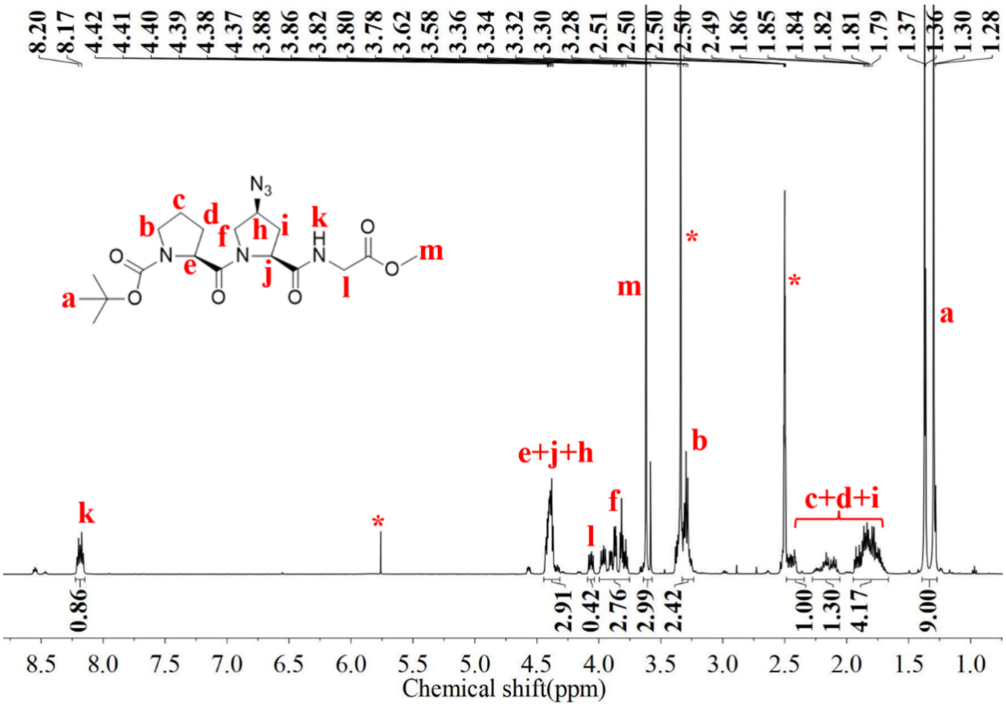

Figure S3. ^{1}H NMR spectrum of **Boc-PO(N_3)G-OMe** in *d*6-DMSO. The solvent peak is marked with asterisk (*).

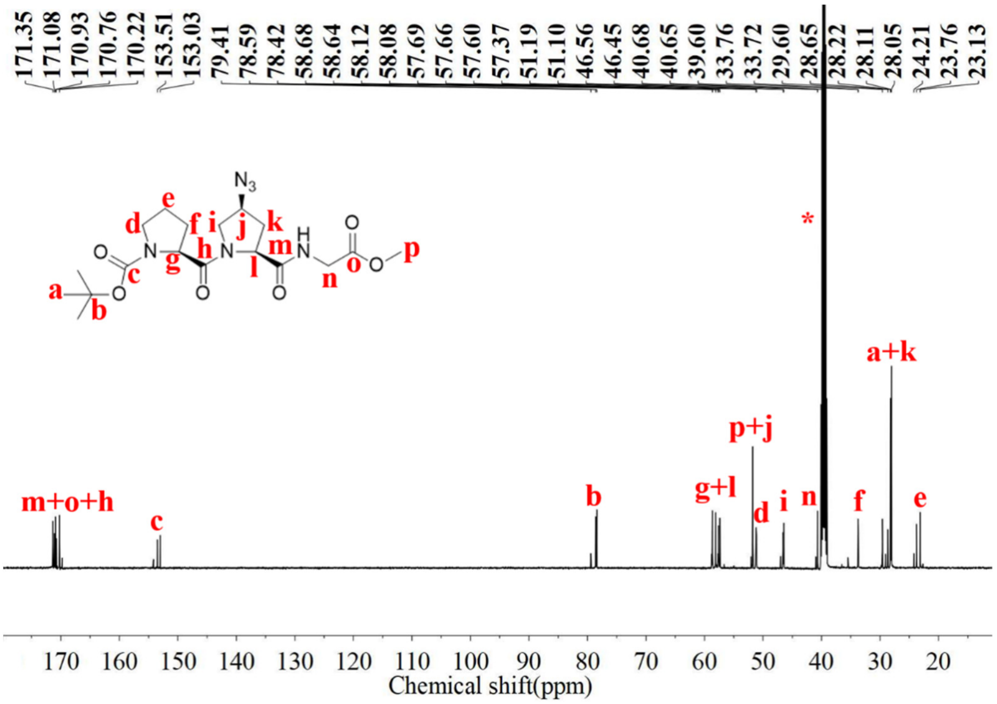

Figure S4.^{13}C NMR spectrum of **Boc-PO(N_3)G-OMe** in *d*6-DMSO. The solvent peak is marked with asterisk (*).

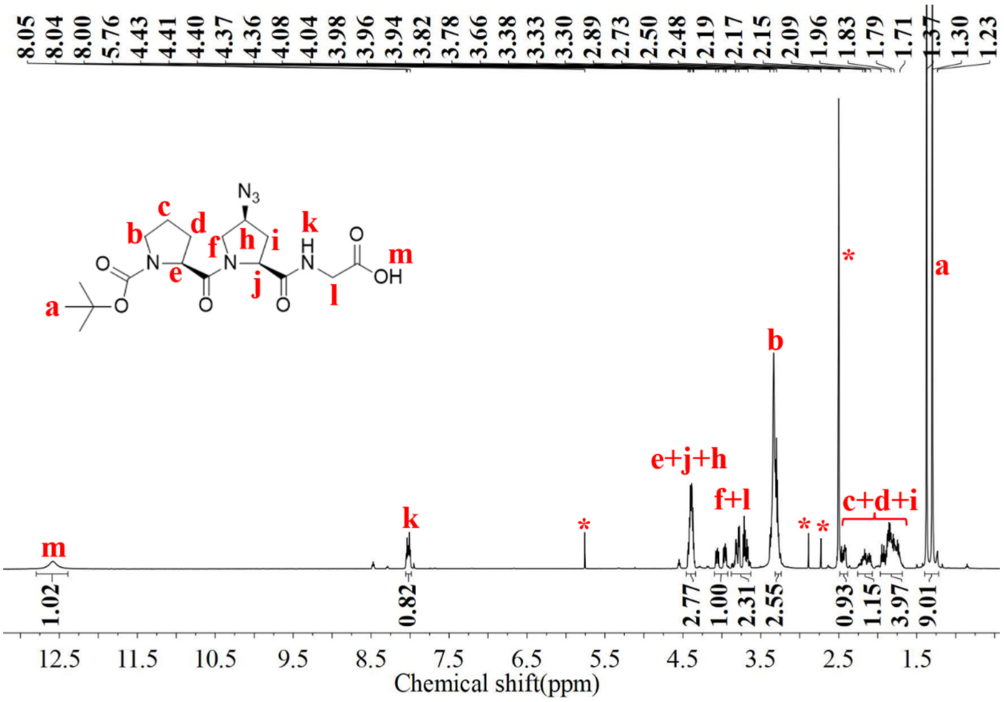

Figure S5. ^{1}H NMR spectrum of **Boc-PO(N$_3$)G-OH** in d_6-DMSO. The solvent peak is marked with asterisk (*).

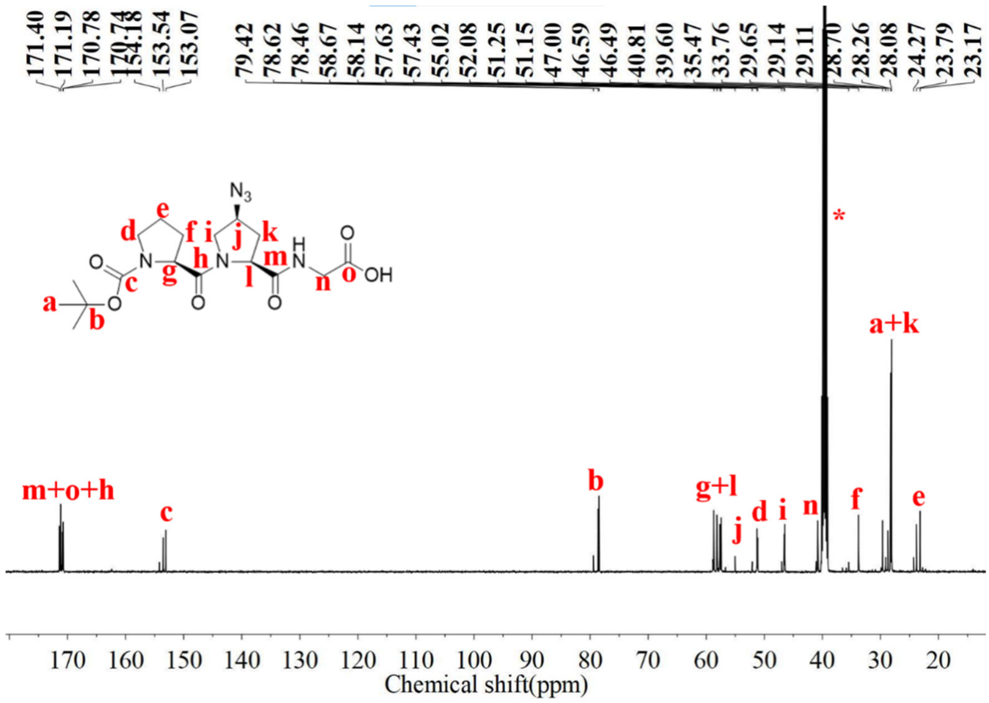

Figure S6. ^{13}C NMR spectrum of **Boc-PO(N$_3$)G-OH** in d_6-DMSO. The solvent peak is marked with asterisk (*).

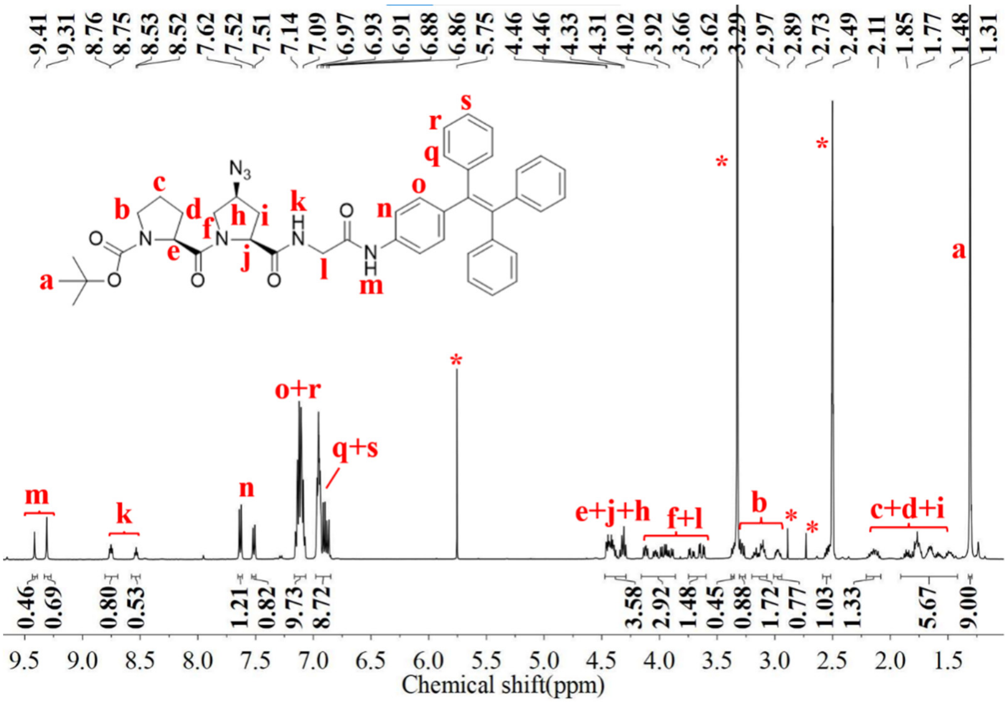

Figure S7. ^{1}H NMR spectrum of **Boc-PO(N_3)G-TPE** in d_6-DMSO. The solvent peak is marked with asterisk (*).

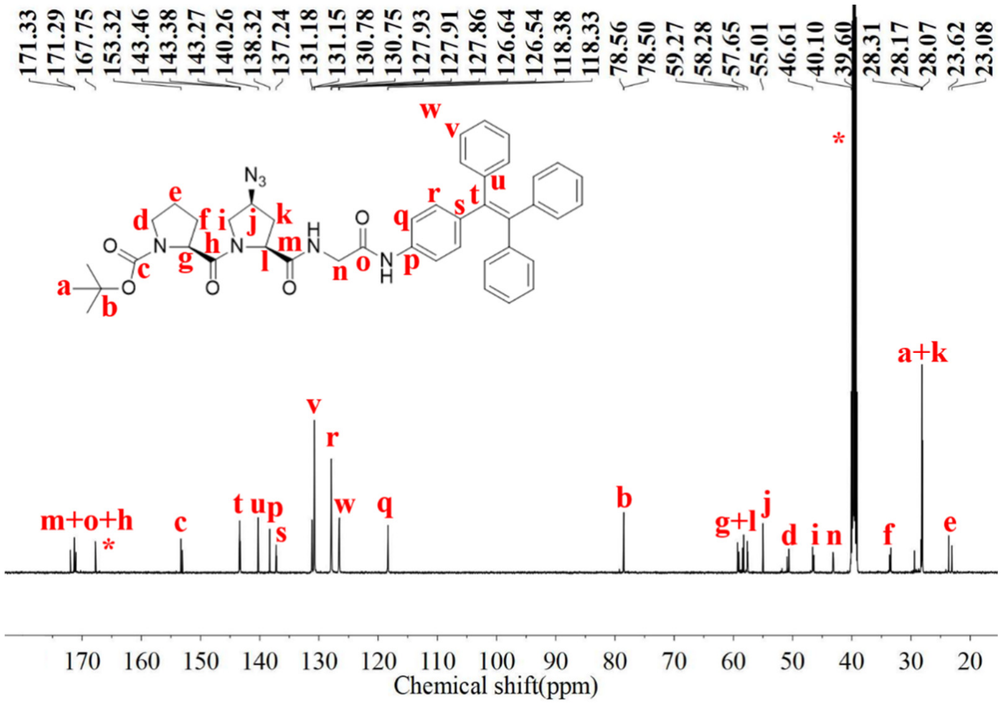

Figure S8. ^{13}C NMR spectrum of **Boc-PO(N_3)G-TPE** in d_6-DMSO. The solvent peak is marked with asterisk (*).

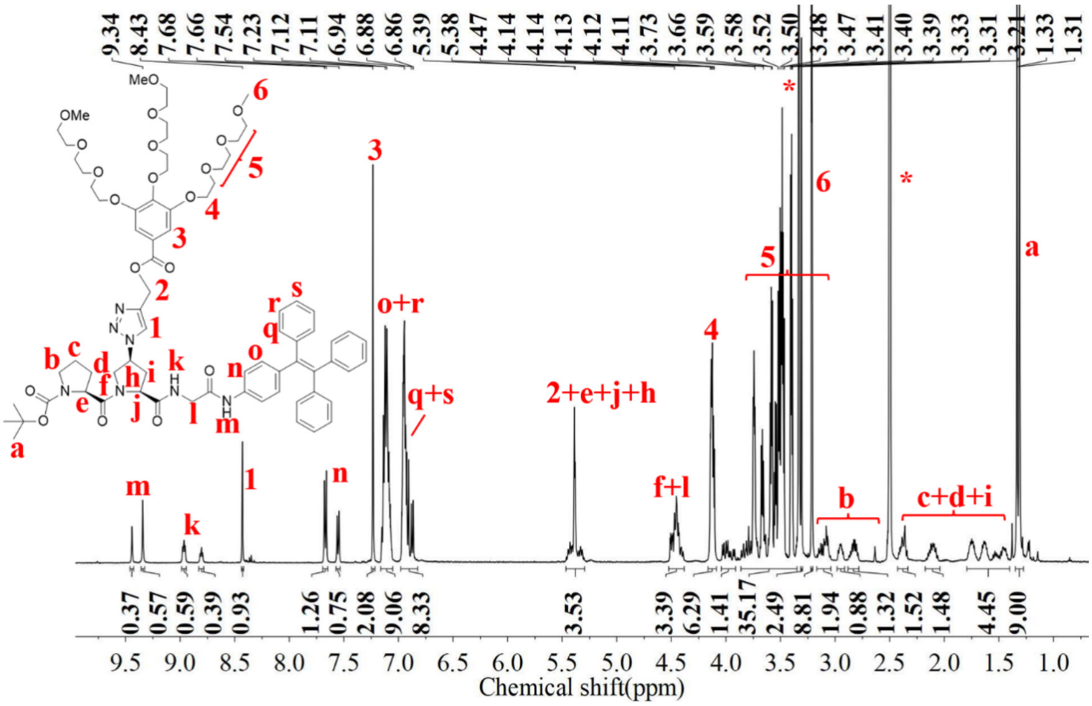

Figure S9. ^{1}H NMR spectrum of **Boc-PO(Me)G-TPE** in d_6-DMSO. The solvent peak is marked with asterisk (*).

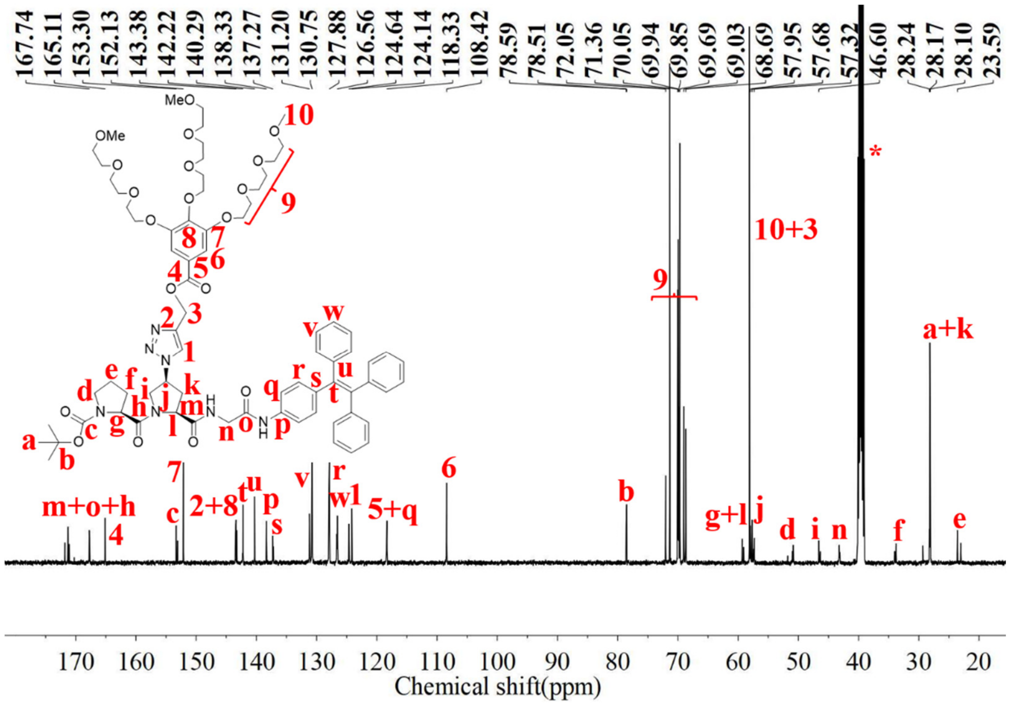

Figure S10. ^{13}C NMR spectrum of **Boc-PO(Me)G-TPE** in d_6-DMSO. The solvent peak is marked with asterisk (*).

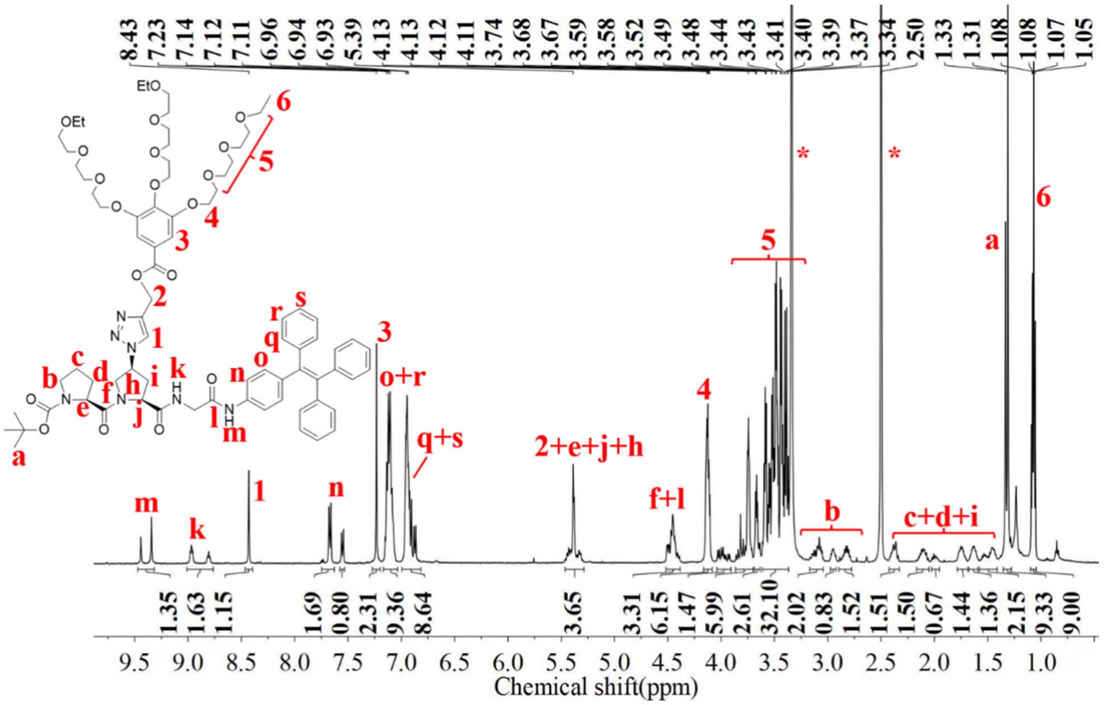

Figure S11. ^{1}H NMR spectrum of **Boc-PO(Et)G-TPE** in d_6-DMSO. The solvent peak is marked with asterisk (*).

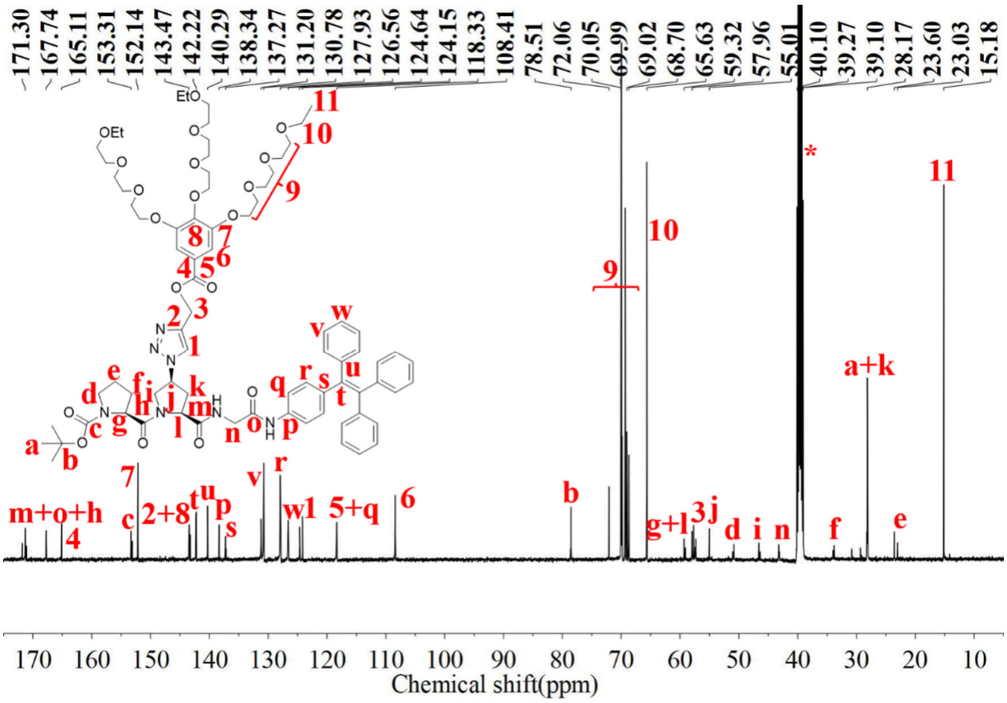

Figure S12. ^{13}C NMR spectrum of **Boc-PO(Et)G-TPE** in d_6-DMSO. The solvent peak is marked with asterisk (*).

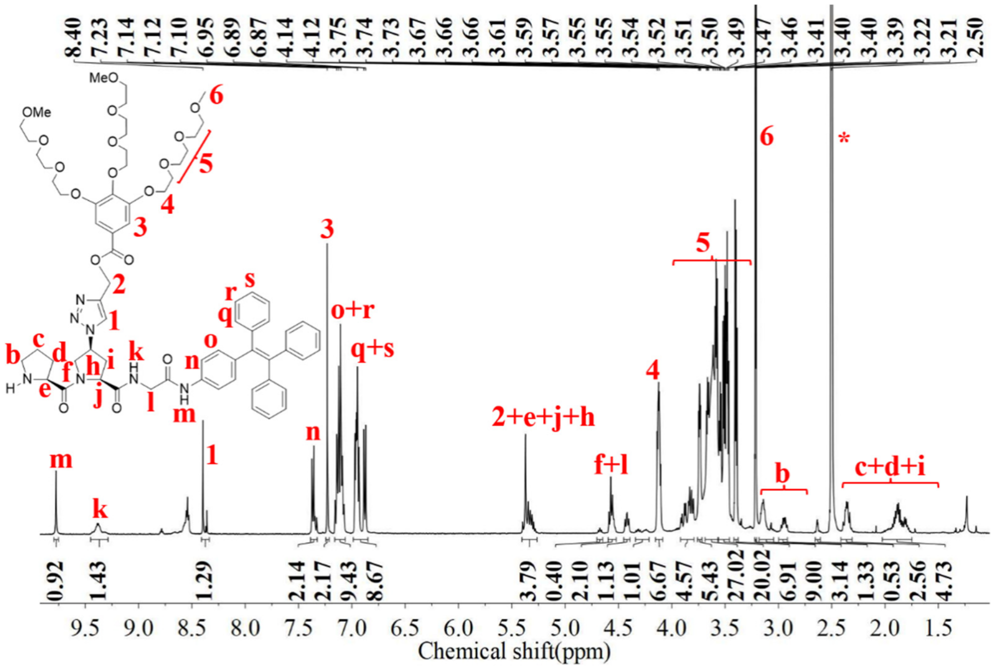

Figure S13. ^{1}H NMR spectrum of **H-PO(Me)G-TPE** in d_6-DMSO. The solvent peak is marked with asterisk (*).

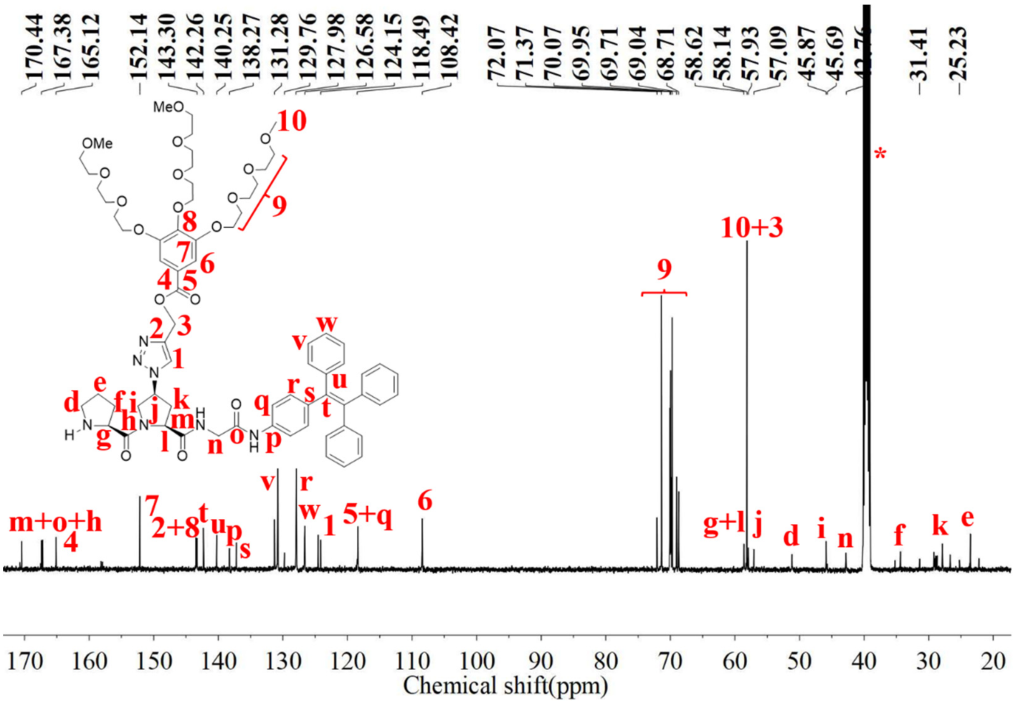

Figure S14. ^{13}C NMR spectrum of **H-PO(Me)G-TPE** in d_6-DMSO. The solvent peak is marked with asterisk (*).

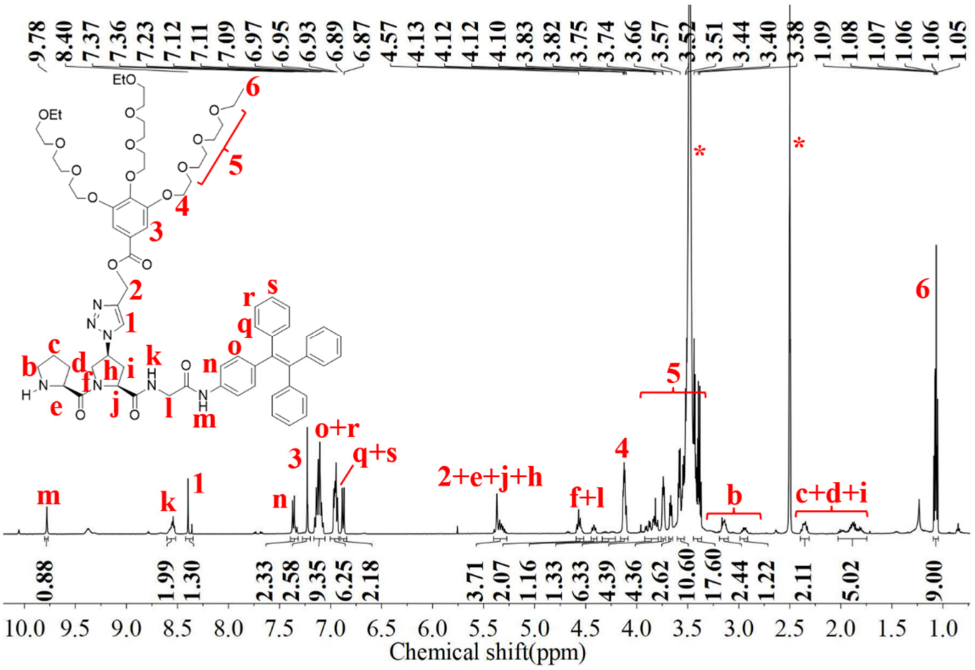

Figure S15. ^{1}H NMR spectrum of **H-PO(Et)G-TPE** in d_6-DMSO. The solvent peak is marked with asterisk (*).

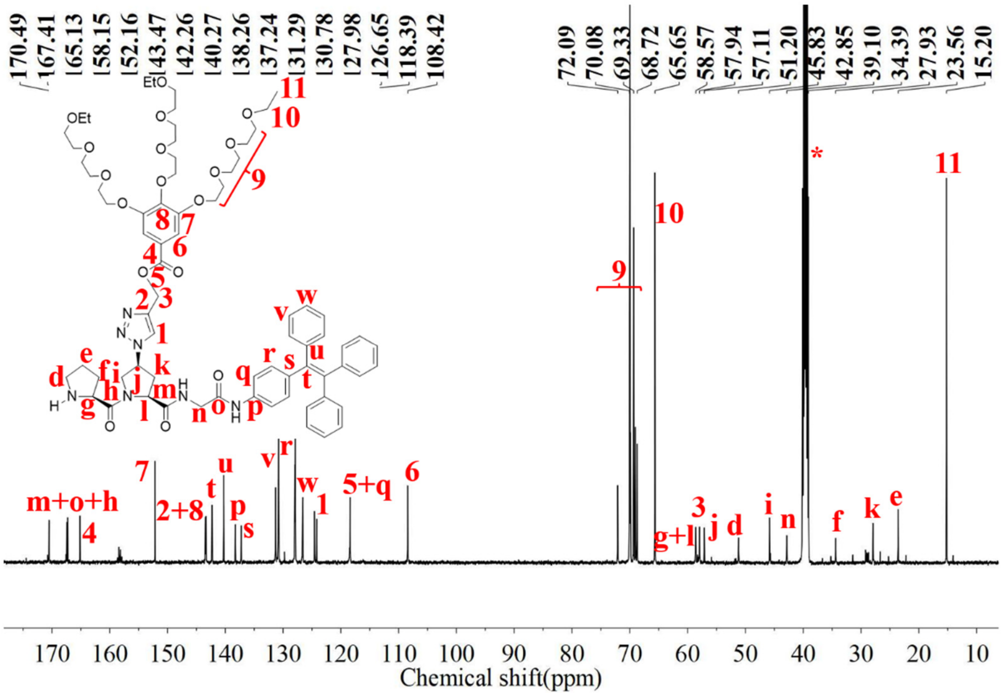

Figure S16. ^{13}C NMR spectrum of **H-PO(Et)G-TPE** in d_6-DMSO. The solvent peak is marked with asterisk (*).

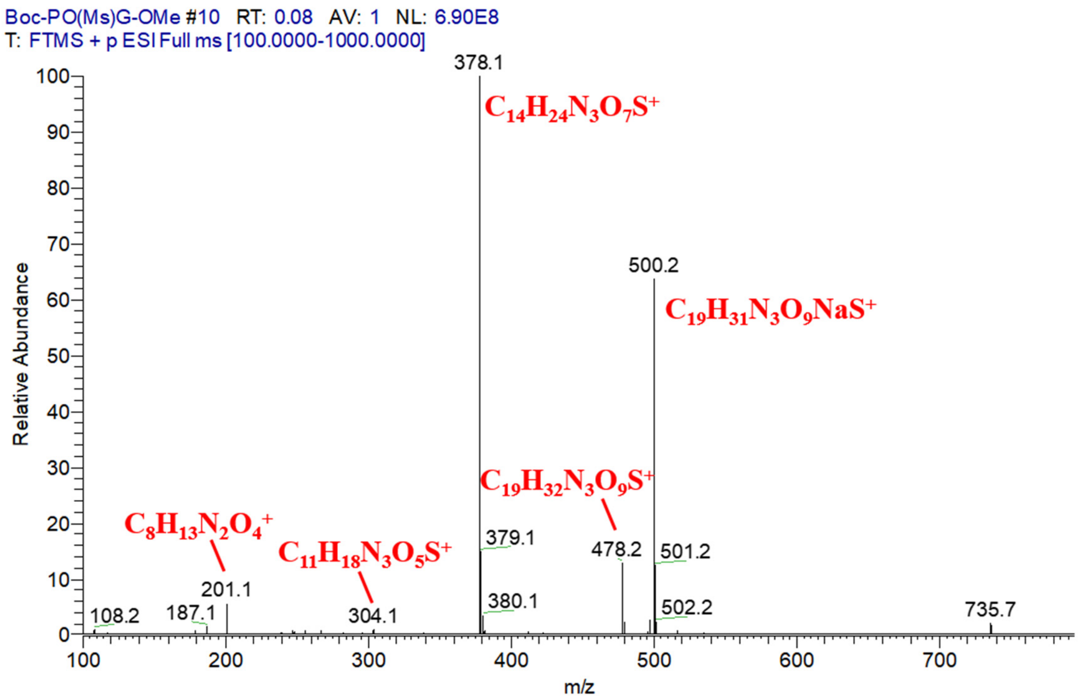

Figure S17. ESI-MS (pos. mode, DCM) of **Boc-PO(Ms)G-OMe**.

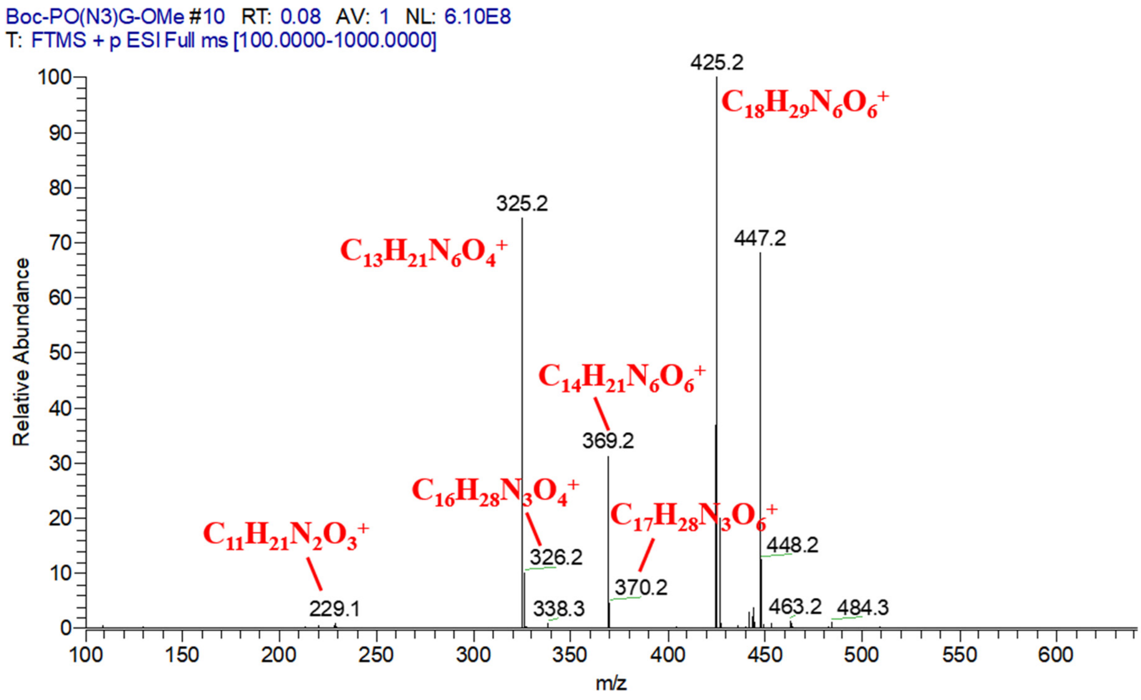

Figure S18. ESI-MS (pos. mode, DCM) of **Boc-PO(N$_3$)G-OMe**.

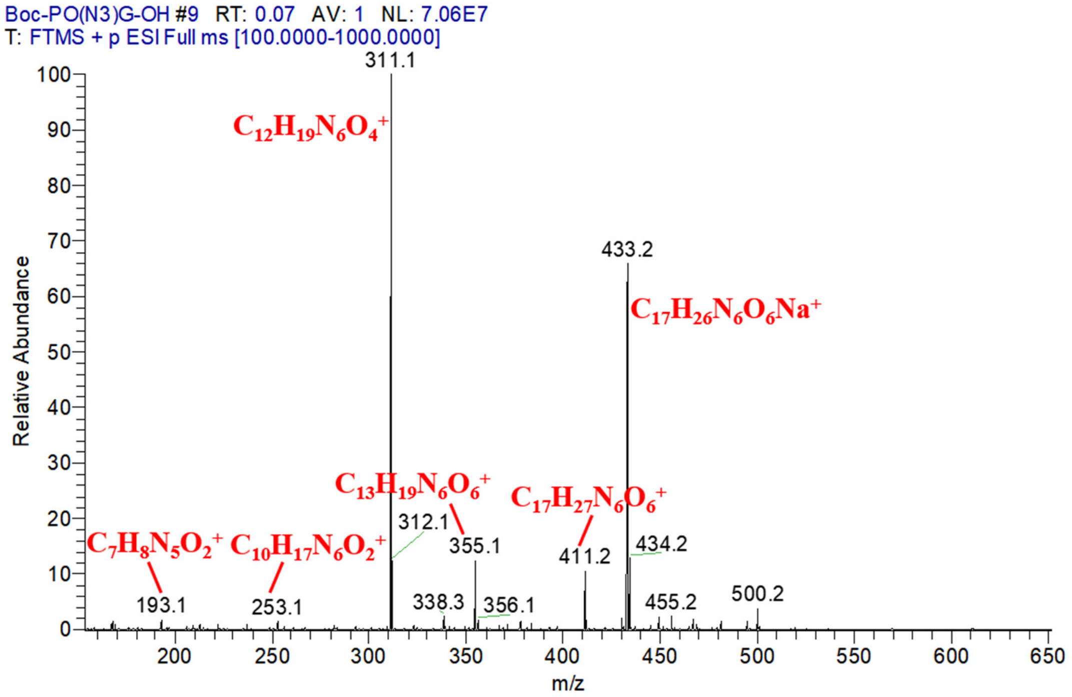

Figure S19. ESI-MS (pos. mode, DCM) of **Boc-PO(N3)G-OH**.

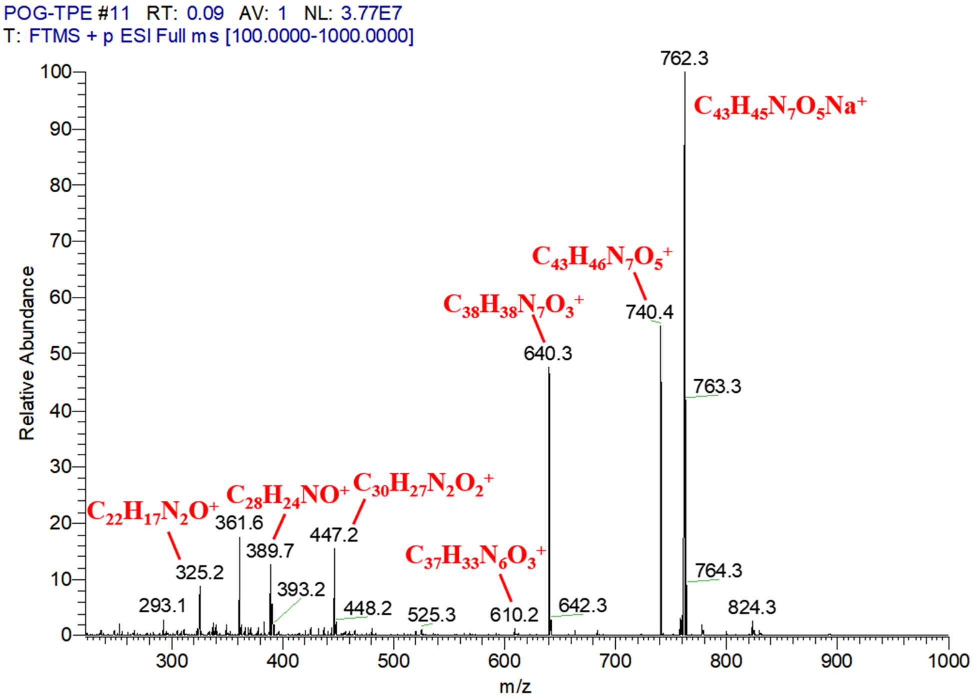

Figure S20. ESI-MS (pos. mode, DCM) of **Boc-PO(N3)G-TPE**.

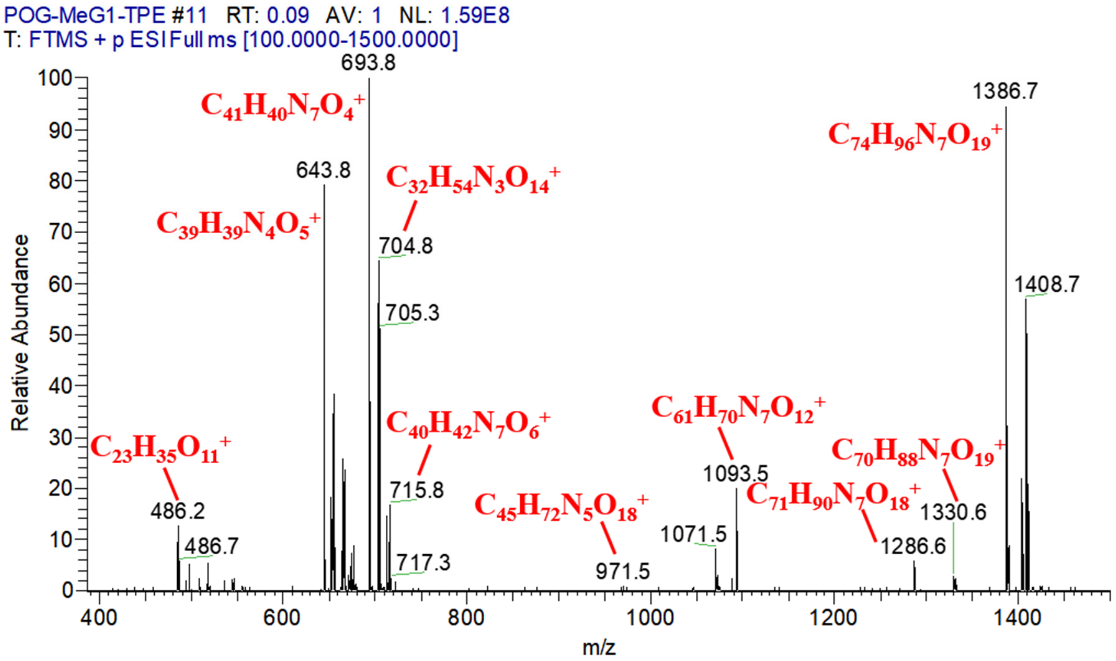

Figure S21. ESI-MS (pos. mode, DCM) of **Boc-PO(Me)G-TPE**.

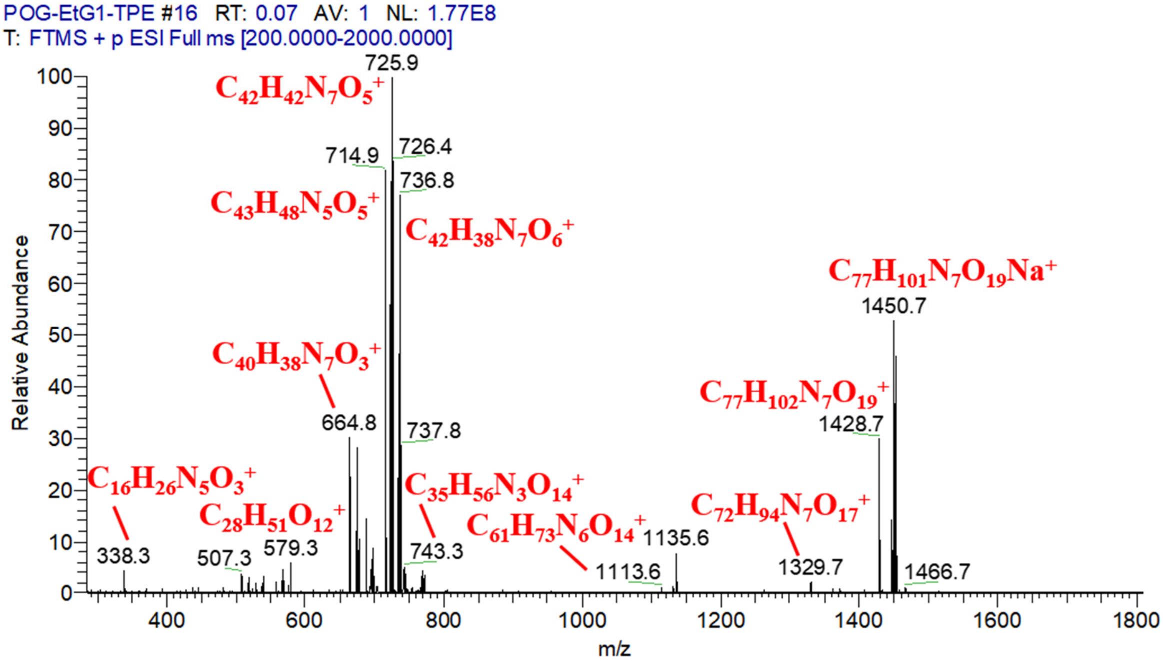

Figure S22. ESI-MS (pos. mode, DCM) of **Boc-PO(Et)G-TPE**.

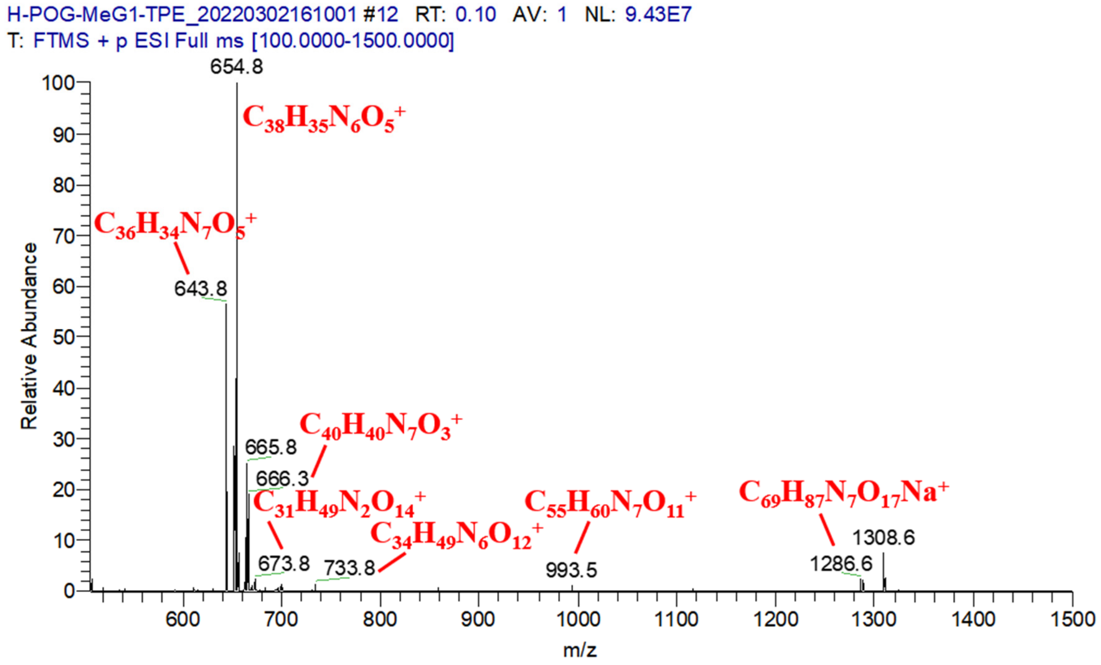

Figure S23. ESI-MS (pos. mode, DCM) of **H-PO(Me)G-TPE**.

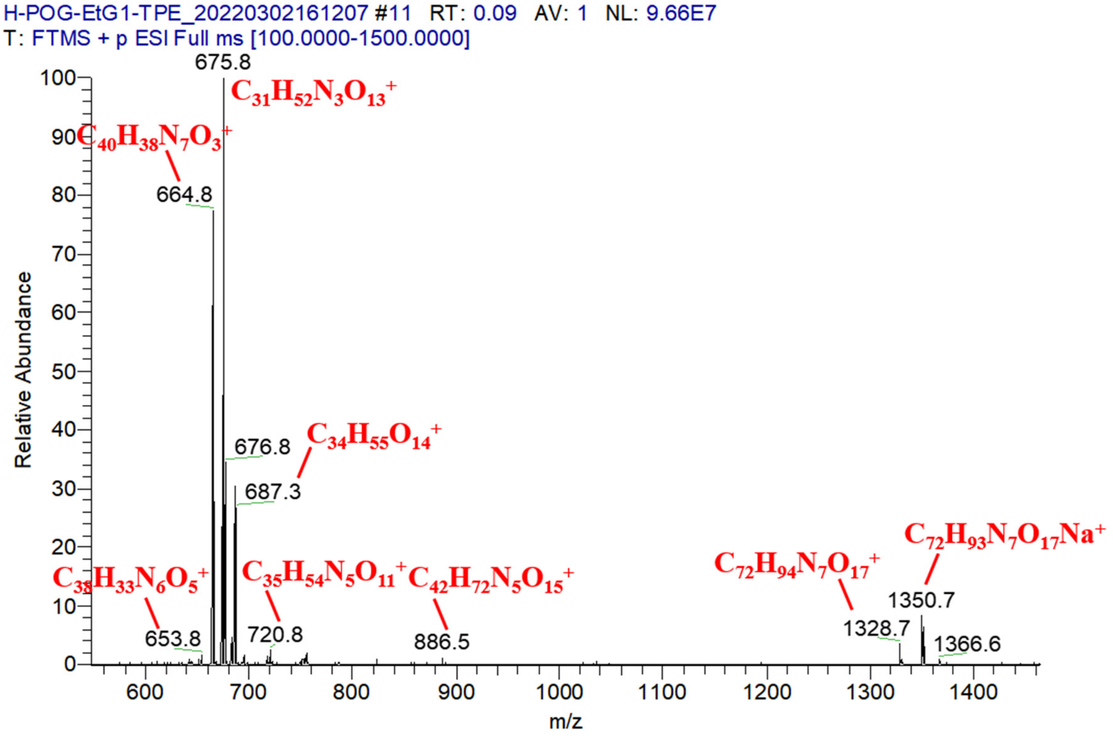

Figure S24. ESI-MS (pos. mode, DCM) of **H-PO(Et)G-TPE**.

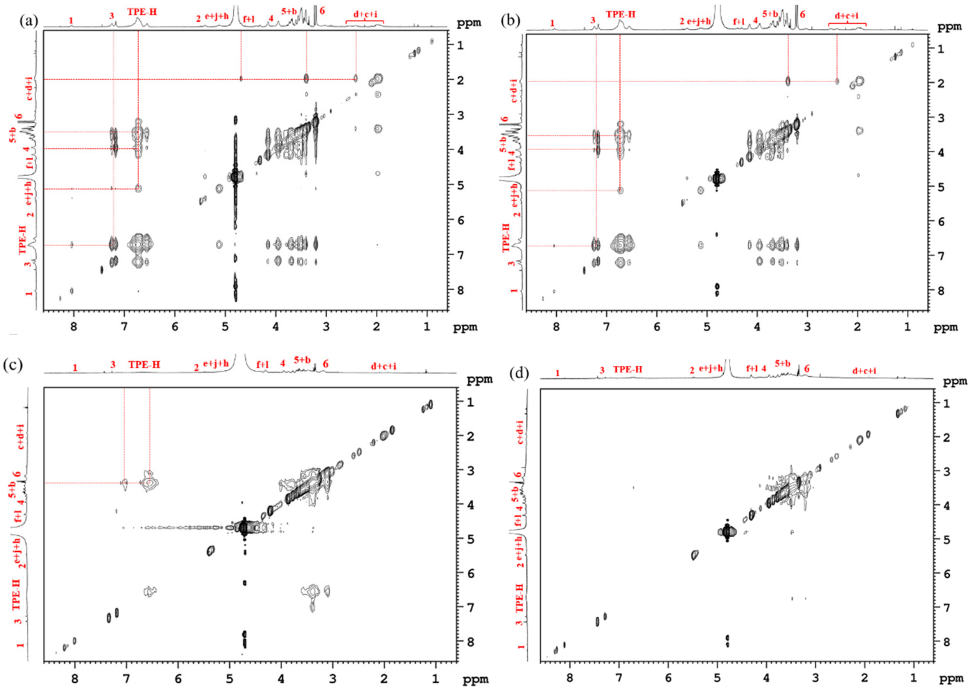

Figure S25. NOESY spectra of **H-PO(Me)G-TPE** in D2O at pH 4 (a), pH 5 (b), pH 6 (c), and pH 7 (d). T = 25 °C. C = 1.0 mg·mL^{-1}.

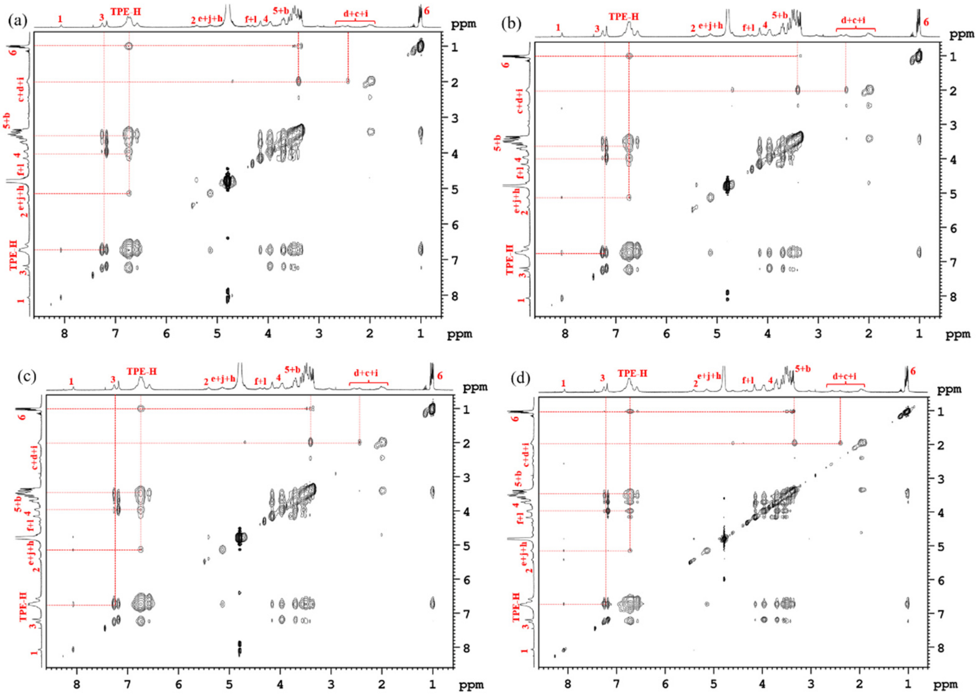

Figure S26. NOESY spectra of **H-PO(Et)G-TPE** in D2O at pH 1 (a), pH 3 (b), pH 5 (c), and pH 6 (d). T = 25 °C. C = 1.0 mg·mL^{-1}.

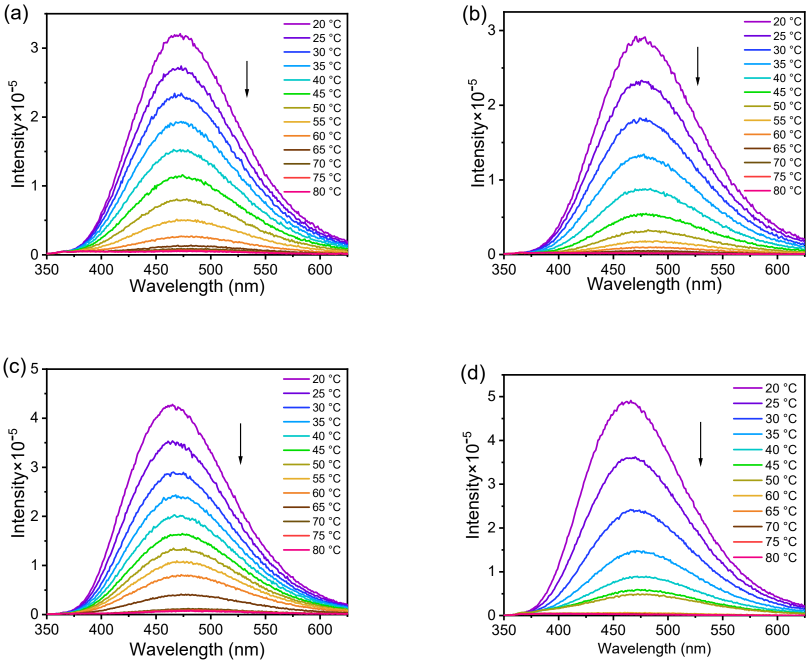

Figure S27. Temperature-varied FL spectra of **H-PO(Me)G-TPE** in aqueous solutions at pH 4 (a), pH 5 (b), pH 6 (c), and pH 7 (d). C = 0.2 mg·mL^{-1}, λ_{ex} = 325 nm.

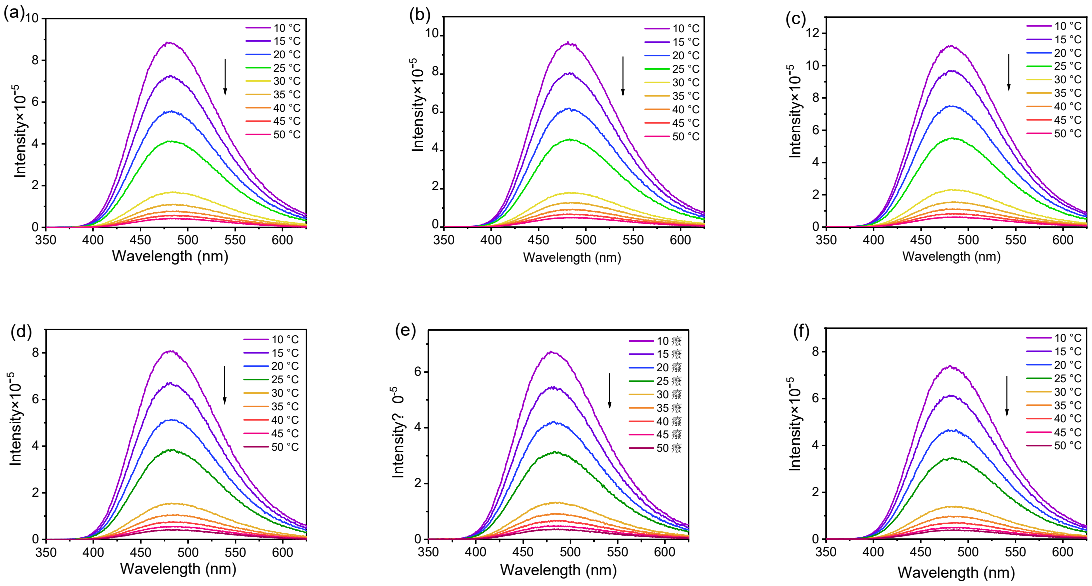

Figure S28. Temperature-varied FL spectra of **H-PO(Et)G-TPE** in aqueous solution at pH 1 (a), pH 2 (b), pH 3 (c), pH 4 (d), pH 5 (e), and pH 6 (f). C = 0.2 mg·mL^{-1}, λ_{ex} = 325 nm.

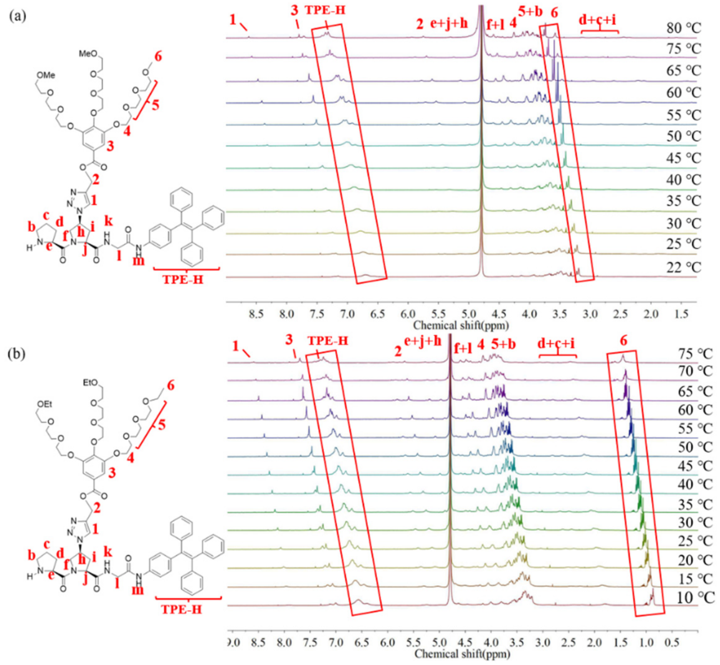

Figure S29. ^{1}H NMR spectra of **H-PO(Me)G-TPE** (a) and **H-PO(Et)G-TPE** (b) in D_2O at different temperature. pH = 6, C = 1.0 mg·mL^{-1}.

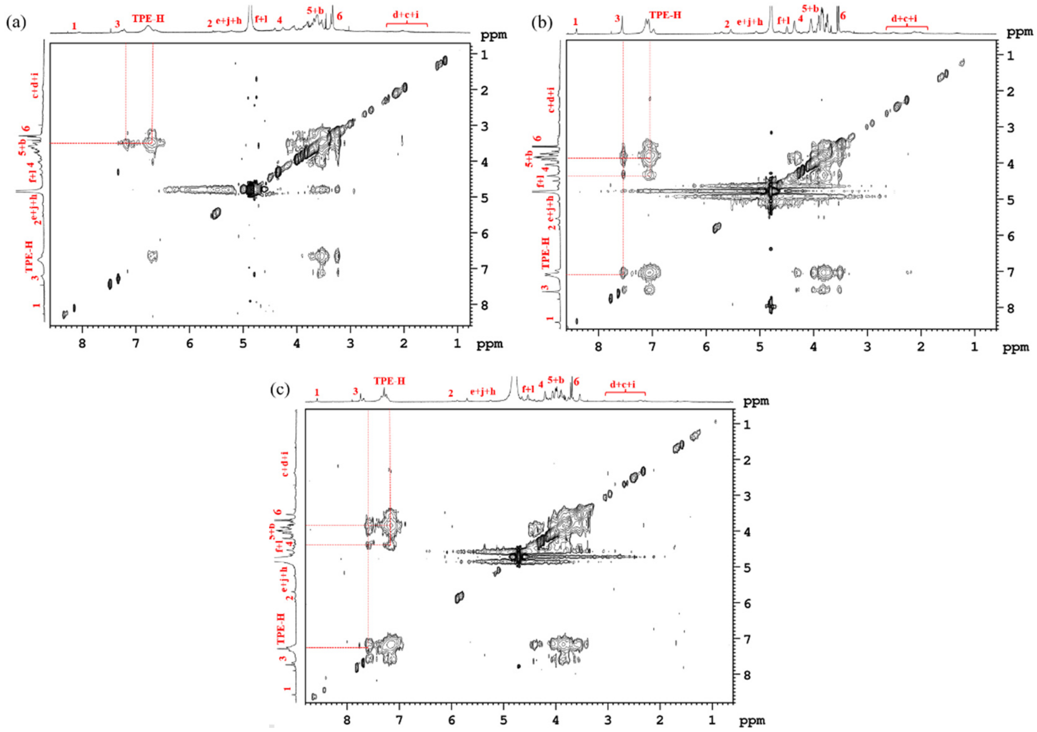

Figure S30. NOESY spectra of **H-PO(Me)G-TPE** in D_2O at 25 °C (a), 60 °C (b), and 75 °C (c). pH = 6. C = 1.0 mg·mL^{-1}.

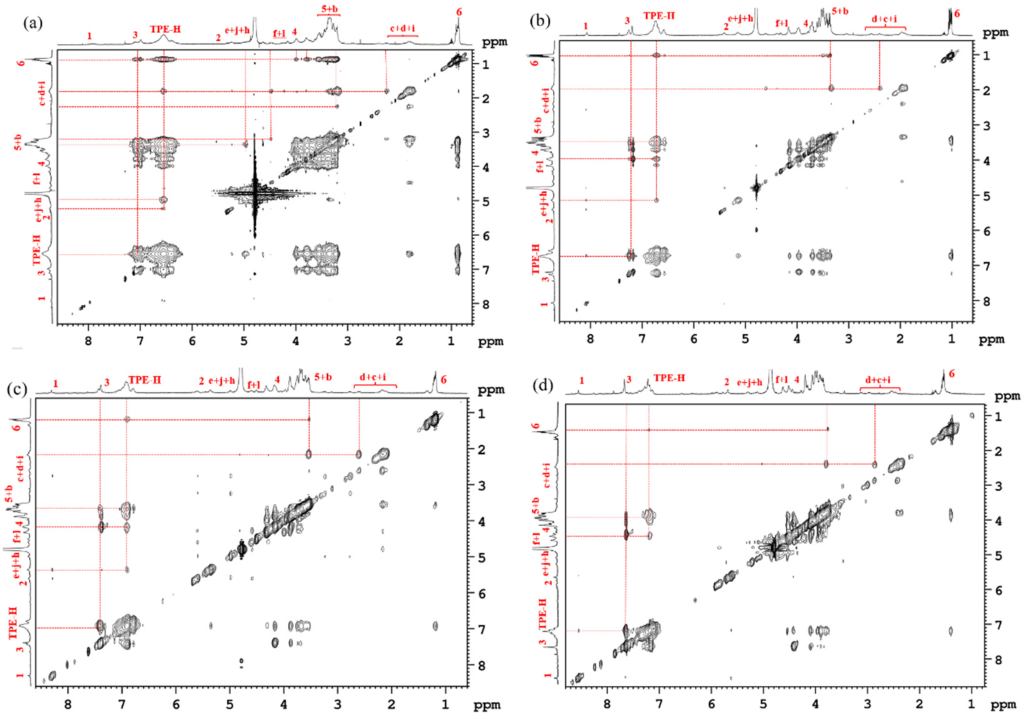

Figure S31. NOESY spectra of **H-PO(Et)G-TPE** in D$_2$O at 10 °C (a), 25 °C (b), 45 °C (c), and 70 °C (d). pH = 6. C = 1.0 mg·mL^{-1}.

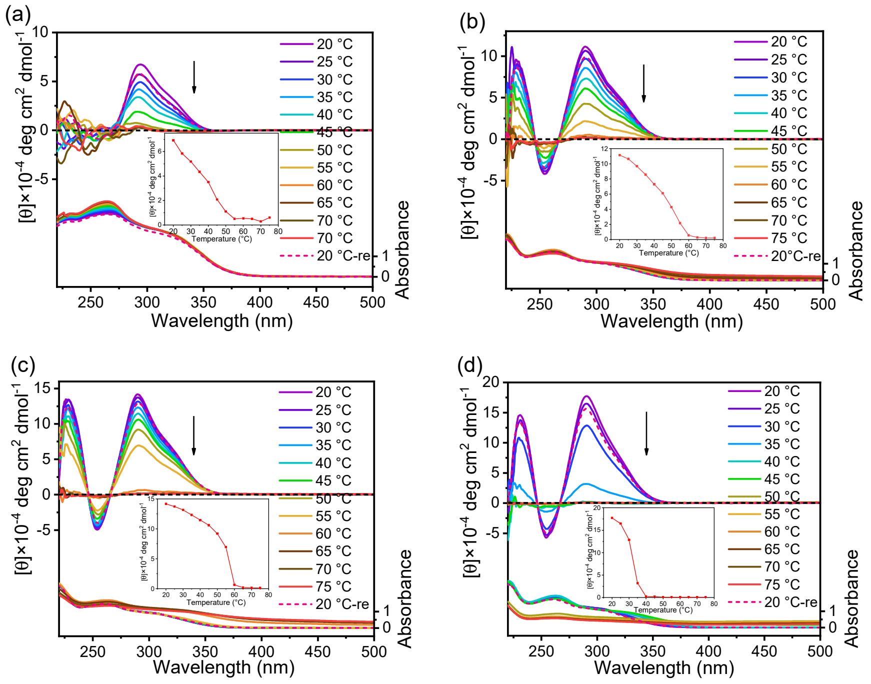

Figure S32. Temperature-varied CD and UV spectra of the assembly from **H-PO(Me)G-TPE** in aqueous solutions at pH 4 (a), pH 5 (b), pH 6 (c), and pH 7 (d). C = 1.0 mg·mL^{-1}. Heating rate = 2.0 ºC. Insets: plots of $[\theta]_{300}$ against temperatures.

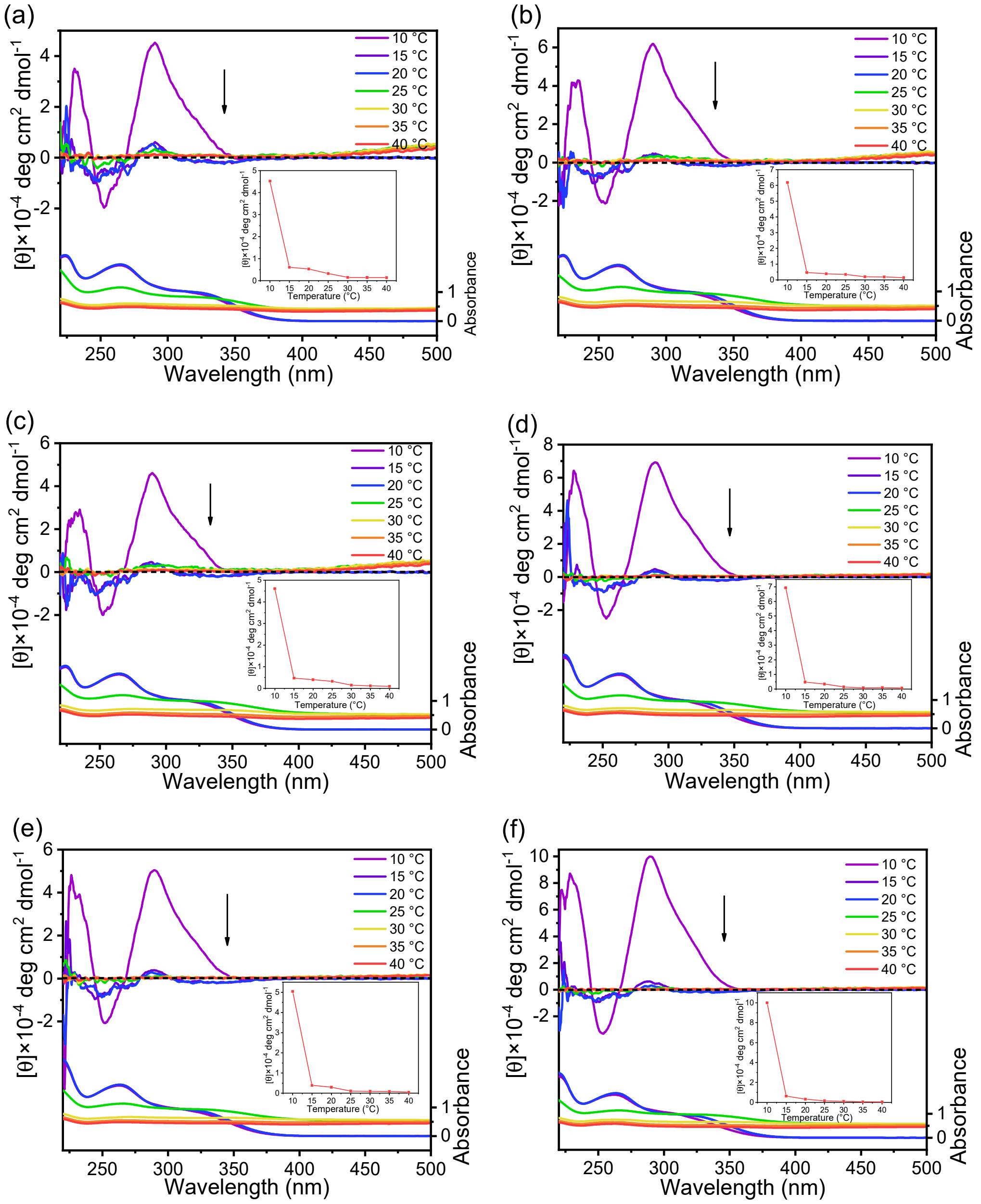

Figure S33. Temperature-varied CD and UV spectra of the assembly from **H-PO(Et)G-TPE** in aqueous solutions at pH 1 (a), pH 2 (b), pH 3 (c), pH 4 (d), pH 5 (e), and pH 6 (f). C = 1.0 mg·mL^{-1}. Heating rate = 2.0 ºC. Insets: plots of $[\theta]_{300}$ against temperatures.
